# Control of Protein Activity and Cell Fate Specification via Light-Mediated Nuclear Translocation

**DOI:** 10.1371/journal.pone.0128443

**Published:** 2015-06-17

**Authors:** Hayretin Yumerefendi, Daniel J. Dickinson, Hui Wang, Seth P. Zimmerman, James E. Bear, Bob Goldstein, Klaus Hahn, Brian Kuhlman

**Affiliations:** 1 Department of Biochemistry and Biophysics, University of North Carolina, Chapel Hill, North Carolina, United States of America; 2 Department of Pharmacology, University of North Carolina, Chapel Hill, North Carolina, United States of America; 3 Department of Biology, University of North Carolina, Chapel Hill, North Carolina, United States of America; 4 Department of Cell Biology & Physiology, University of North Carolina, Chapel Hill, North Carolina, United States of America; 5 Lineberger Comprehensive Cancer Center, University of North Carolina, Chapel Hill, North Carolina, United States of America; University of Toronto, CANADA

## Abstract

Light-activatable proteins allow precise spatial and temporal control of biological processes in living cells and animals. Several approaches have been developed for controlling protein localization with light, including the conditional inhibition of a nuclear localization signal (NLS) with the Light Oxygen Voltage (AsLOV2) domain of phototropin 1 from *Avena sativa*. In the dark, the switch adopts a closed conformation that sterically blocks the NLS motif. Upon activation with blue light the C-terminus of the protein unfolds, freeing the NLS to direct the protein to the nucleus. A previous study showed that this approach can be used to control the localization and activity of proteins in mammalian tissue culture cells. Here, we extend this result by characterizing the binding properties of a LOV/NLS switch and demonstrating that it can be used to control gene transcription in yeast. Additionally, we show that the switch, referred to as LANS (light-activated nuclear shuttle), functions in the *C*. *elegans* embryo and allows for control of nuclear localization in individual cells. By inserting LANS into the *C*. *elegans lin-1* locus using Cas9-triggered homologous recombination, we demonstrated control of cell fate via light-dependent manipulation of a native transcription factor. We conclude that LANS can be a valuable experimental method for spatial and temporal control of nuclear localization *in vivo*.

## Introduction

Many biological processes rely on the precise spatial segregation of macromolecules within a living cell. Regulated compartmental partitioning is a common mechanism for controlling gene expression through sequestering transcription factors in the cytoplasm [1]. Analogously, inducible nucleocytoplasmic translocation represents a powerful approach to control cell behavior by conditionally removing a protein of interest from the cellular compartment where it is active. We sought to generate a single component, genetically encoded and reversible light-driven nuclear import switch. We hypothesized that it would allow for simple control of genes of interest in a variety of experimental systems, including multicellular organisms.

Protein engineering in combination with small molecules has previously been used to control nucleocytoplasmic translocation. Early examples of experimental tools for conditional nuclear import have used the nuclear hormone receptor Estrogen Receptor α (ERα), which is cytoplasmic until bound to its ligand. This approach was first applied to control the Myc transcription factor [2], and an improved version that makes use of the estrogen receptor antagonist tamoxifen has been widely used for inducing site-specific recombination with the Cre recombinase (Cre-ER^T^) [3, 4]. Anchor-Away is a two-component system that works by sequestering a protein of interest in the cytosol via rapamycin-dependent heterodimerization between FKBP12 and the FRB domain of human mTOR kinase. One is fused to a ribosomal protein and the other to the protein of interest [5]. These approaches rely on the small chemical molecules tamoxifen and rapamycin for induction. Chemical induction requires that small molecules enter cells and biological tissue, has limited reversibility and lacks fine spatial control in an organism.

Optogenetic tools are minimally invasive, allow for subcellular spatial control, and have reversible, rapid and adjustable effects on time scales from milliseconds to hours [6]. Transparent model organisms such as the nematode *C*. *elegans*, the fly *D*. *melanogaster* and the zebrafish *D*. *rerio* are especially well suited for optogenetics, and photoactivatable proteins have enabled discoveries in these systems unattainable with conventional techniques [7–9]. Light-activated control of nuclear import represents a powerful and potentially general way of controlling multiple cellular functions.

Deiters and co-workers controlled protein localization by incorporating a photoactive amino acid in a nuclear localization signal so that it could only interact with the nuclear import machinery when the chemical moiety was removed via irradiation with UV light [10, 11]. This approach is not reversible and requires the bioavailability of a non-natural amino acid. Equivalent to the Anchor-Away technique is a recently developed organelle targeting system that uses the red light mediated interaction between phytochrome B (PhyB) and phytochrome-interacting factor 6 (PIF6) [12]. The association and dissociation kinetics of this system are rapid and it has been used to study the effects of the mitotic cyclin Clb2 in nuclear fission and spindle stabilization in yeast. However, the requirement for a non-natural cofactor (PCB) presents an obstacle to the use of this system in living animals.

Very recently the first fully optogenetic tool for the control of nuclear import was reported by Niopek and co-workers [13]. The engineered switch makes use of the LOV2 domain from *Avena Sativa* (AsLOV2). When activated with blue light, the AsLOV2 domain undergoes a conformational change and the C-terminal Jα helix unfolds. To control nuclear localization, a NLS motif was embedded at the end of the Jα helix so that it is sterically hindered from binding the nuclear import machinery when the AsLOV2 is in its closed, dark-state conformation. Upon activation with light, the NLS becomes accessible and the protein is imported to the nucleus. To make the switch reversible, a constitutive NES was added to direct the protein to the cytoplasm when the NLS motif is hidden in the dark state. It was shown that it was important to tune the relative strengths of the NLS and NES motifs to maximize the dynamic range of the switch. To demonstrate functional activity, the switch was used to control transcription of a reporter gene in mammalian cells.

Here, we confirm and extend the findings of Niopek et al. [13] and present the design, engineering and application of a Light-Activated Nuclear Shuttle (LANS) that also makes use of the AsLOV2 domain to cage a NLS motif. We directly show that LANS functions by regulating its binding affinity to variants of importin α. LANS allows for robust control of transcription in yeast and exhibits fast blue light-induced nuclear import as well as dark cytoplasmic reversion in a variety of mammalian tissue culture cells. CRISPR/Cas9-mediated insertion of LANS into the *lin-1* gene of *C*. *elegans* conferred light dependence on an endogenous cellular transcription factor, allowing optogenetic control of vulval cell fate specification in living animals.

## Results

### Design of a light-conditioned nuclear localization signal

**Fig 1:**
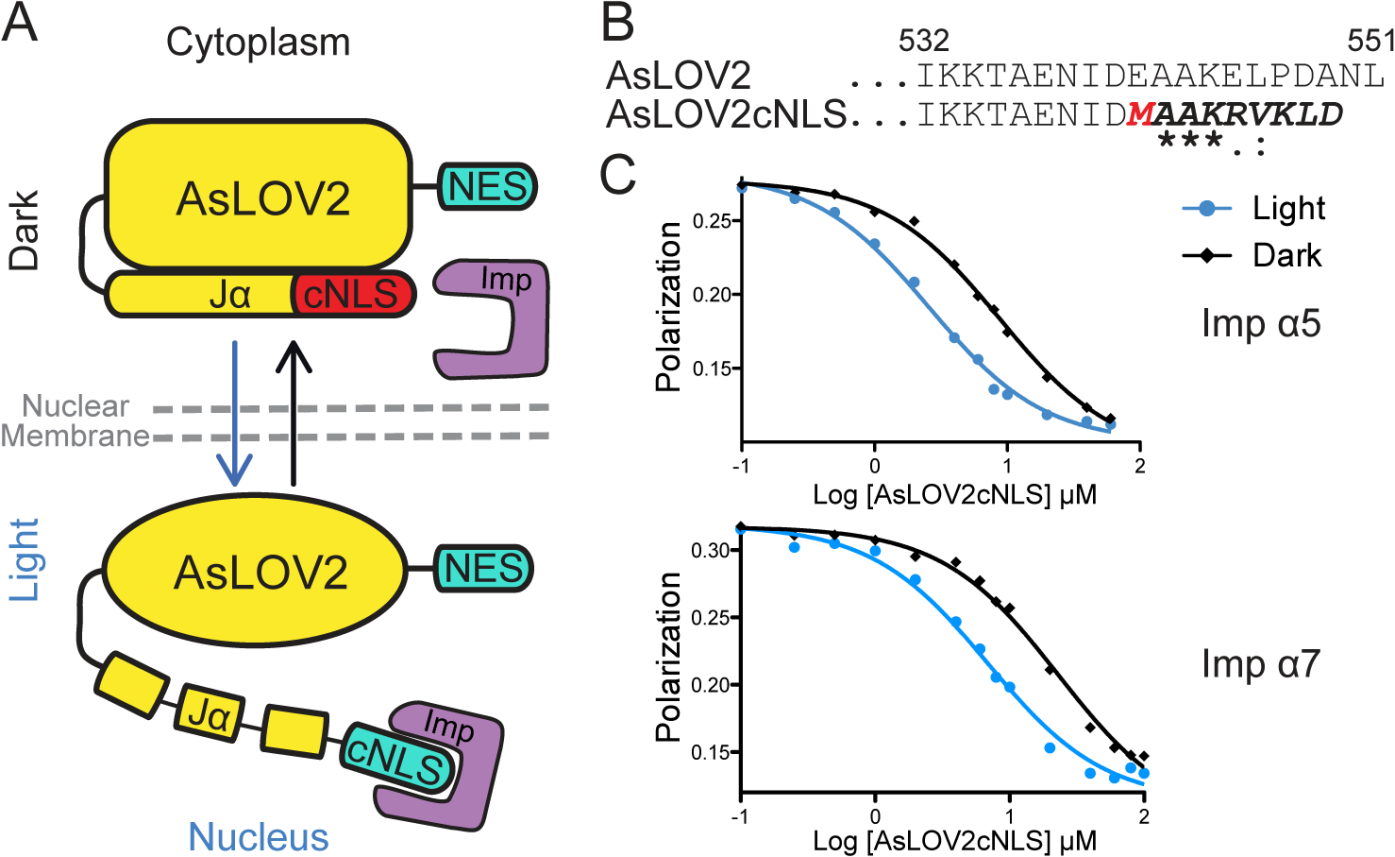
Design and biophysical characterization of light conditioned nuclear localization signal. (A) Schematic of the Light Activated Nuclear Shuttle (LANS) design for light activated nuclear import (B) Sequence alignment of the wild type AsLOV2 and the designed AsLOV2cNLS (sequence identity and homology is marked according to CLUSTALW scheme). (C) Fluorescence polarization competitive binding assay of AsLOV2cNLS against human importin α5 and importin α7.

To control nuclear import with light we engineered a conditional Nuclear Localization Signal (cNLS) that would be allosterically blocked in the dark but available for binding to importin in the light (Fig 1A). Previously, the AsLOV2 domain from *Avena sativa* has been successfully used to control the binding of short, linear sequence epitopes [14–16] and does not contain an endogenous nuclear localization signal. Therefore, to generate an allosterically caged NLS, we first attached the human Myc NLS at the end of the AsLOV2 domain after residue 546, aligning the proline residue from the NLS sequence to the proline residue of AsLOV2. This fusion protein (AsLOV2cMyc) bound importin α5 with low nanomolar affinity and showed no light-dependence (supplemental). Next, we decided to embed the Myc NLS further into the Jα helix, aligning the alanine residues present in both sequences (Fig 1B). To eliminate the conserved proline residue at the beginning of the NLS, which could disrupt the helicity of the Jα, we performed design simulations with the modeling program Rosetta using the karyopherin-Myc NLS complex structure (PDB: 1EE4). We allowed only the proline residue of the Myc NLS to vary and identified a favorable methionine substitution (Fig 1B and S1 Fig). Rosetta was then used to build a model of the designed NLS sequence, grafted onto the AsLOV2 structure (PDB: 2v0u) creating AsLOV2cNLS. The designed methionine pointed towards solvent, not clashing with any residues from the AsLOV2 domain, and the remaining hydrophobic residues present in the NLS were well packed against the core PAS domain (S1 Fig).

### The switch binds similarly to two distinct importins

Nuclear import efficiency is directly correlated to the binding affinity of a nuclear localization signal for importin α [17]. To determine the binding affinities of the conditioned NLS for human importin α, we adopted a competition-based fluorescence polarization assay [14]. We expressed the AsLOV2cNLS construct and human importins in bacteria, purified them and measured nanomolar affinities for both importins α5 and α7 under blue light irradiation (250 nM and 340 nM, respectively). Their affinities in the absence of light decreased to 1.5 μM for importin α5, and 2 μM for importin α7, which represented a 6-fold change in light/dark affinities (Fig 1C and S2 Fig). Since efficient nuclear localization requires an importin binding affinity tighter than 1 μM [17], these affinity ranges were predictive of efficient nuclear import upon light stimulus and reduced translocation in its absence. The similar binding affinities to two distinct importin α proteins implies that the switch’s nuclear translocation could be mediated by more than one importin *in vivo*. Lastly, we tested whether the chimeric sequence affects the AsLOV2 photocycle. We observed that the dark reversion rate of the AsLOV2cNLS does not deviate from that of wild type AsLOV2, with an activated state half-life of 29.5 sec (S2 Fig).

### A Nuclear Export Signal is required for the reversible control of nuclear import

**Fig 2:**
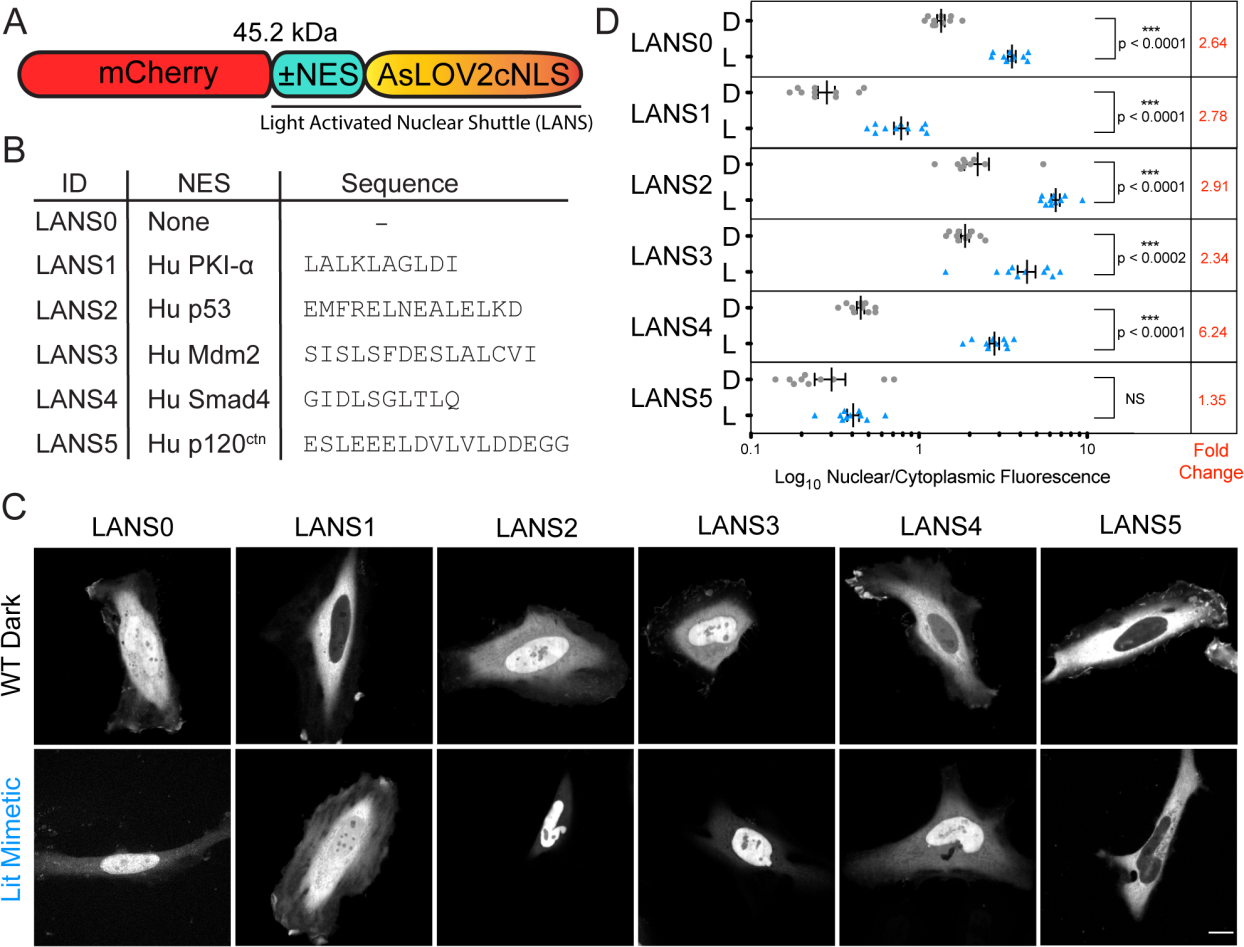
Confocal microscopy of LANS in HeLa cells. (A) Schematic of the LANS constructs (B) List of the nuclear export signals tested. (C) Representative nuclear optical slices of cells used for the quantification of the nuclear/cytoplasmic distribution of the switch (scale bar = 15 μm). (D) Quantification of the effect of the nuclear export signal on the nuclear/cytoplasmic distribution of LANS (D—wild type construct imaged in the dark, L—lit mimetic I539E). Mean is reported ±SEM and statistical significance calculated with unpaired two-tailed t-students test; NS—Not Significant.

To determine whether the AsLOV2cNLS switch could control nuclear localization in live cells, we fused it to mCherry fluorescent protein and observed its sub-cellular distribution in HeLa cells (Fig 2A). In the dark the mCherry fluorescence intensity was almost evenly distributed throughout the cells (Fig 2C top leftmost panel). Incorporating a well-studied AsLOV2 point mutant (I539E) that mimics its lit conformation [18] caused the mCherry signal intensity to concentrate in the nucleus, validating the switch (Fig 2C bottom leftmost panel). Since the nuclear pore complex allows passive diffusion of proteins with molecular weight under 60 kDa [19, 20], we hypothesized that the observed dark state distribution of the 45 kDa mCherry::AsLOV2cNLS protein results from passive diffusion throughout the cell, and that adding a nuclear export sequence (NES) could shift its distribution to the cytoplasm. Hence, we needed to identify the appropriate balance between nuclear import and export signal strength, such that a constitutive NES would be sufficient to remove the switch from the nucleus in the dark but would be overpowered by the strength of the conditional NLS in the light. We screened a panel of five NES sequences that vary in export strength and are derived from human nucleocytoplasmic shuttling proteins: PKI-α [21], p53 [22], Mdm2 [23], Smad4 [24] and p120ctn [25] (Fig 2B). We cloned each of the NES sequences between mCherry and either the wild type or I539E switch variant. For each of the NES variants we quantified the nuclear/cytoplasmic distribution for either the wild type protein in the dark or the lit mimetic, which allowed us to measure steady state distributions without concerns about the precise mode and timing of light stimulation. While the switch with no NES exhibited a 2.64-fold increase in nuclear localization with the lit mimetic, some amount of protein was always present in the nucleus (Dark state = 1.35 ± 0.07 nuclear/cytoplasmic fluorescence, n = 10 and Lit mimetic = 3.57 ± 0.20 nuclear/cytoplasmic fluorescence, n = 10; Fig 2C and 2D). Three of the NES sequences provided lower nuclear fluorescence in the dark, p120ctn, PKI-α, and Smad4 (Fig 2C). However, the NES motif from p120ctn was so strong that nuclear localization was not observed with the lit mutation. The greatest change in nuclear/cytoplasmic fluorescence (6.2 fold) was observed with the NES from Smad4 (Dark state = 0.45 ± 0.02 nuclear/cytoplasmic fluorescence, n = 10 and Lit mimetic = 2.81 ± 0.19 nuclear/cytoplasmic fluorescence, n = 10).

To confirm that exclusion of the constructs from the nucleus required active nuclear export, we treated cells with the Crm1 inhibitor Leptomycin B [26]. Leptomycin B treatment led to loss of nuclear exclusion and restoration of the no-NES construct cellular distribution for all but the p53 and Mdm2 NES (S3 Fig), indicating that the switch constitutively shuttles between the nucleus and the cytoplasm, and confirming that its nuclear export is mediated by Crm1. We therefore named the AsLOV2cNLS switch coupled with an NES the Light Activatible Nuclear Shuttle (LANS). Here, we refer to different LANS constructs by adding a suffix that denotes the NES used: LANS0 carries no NES, while LANS1-5 use the nuclear export signals of PKI-α, p53, Mdm2, Smad4 and p120ctn, respectively (Fig 2B). LANS remains functional when short peptide or large globular proteins are fused to its C-terminus (S3 Fig).

### LANS is imported and exported within minutes in a variety of mammalian cell cultures

**Fig 3:**
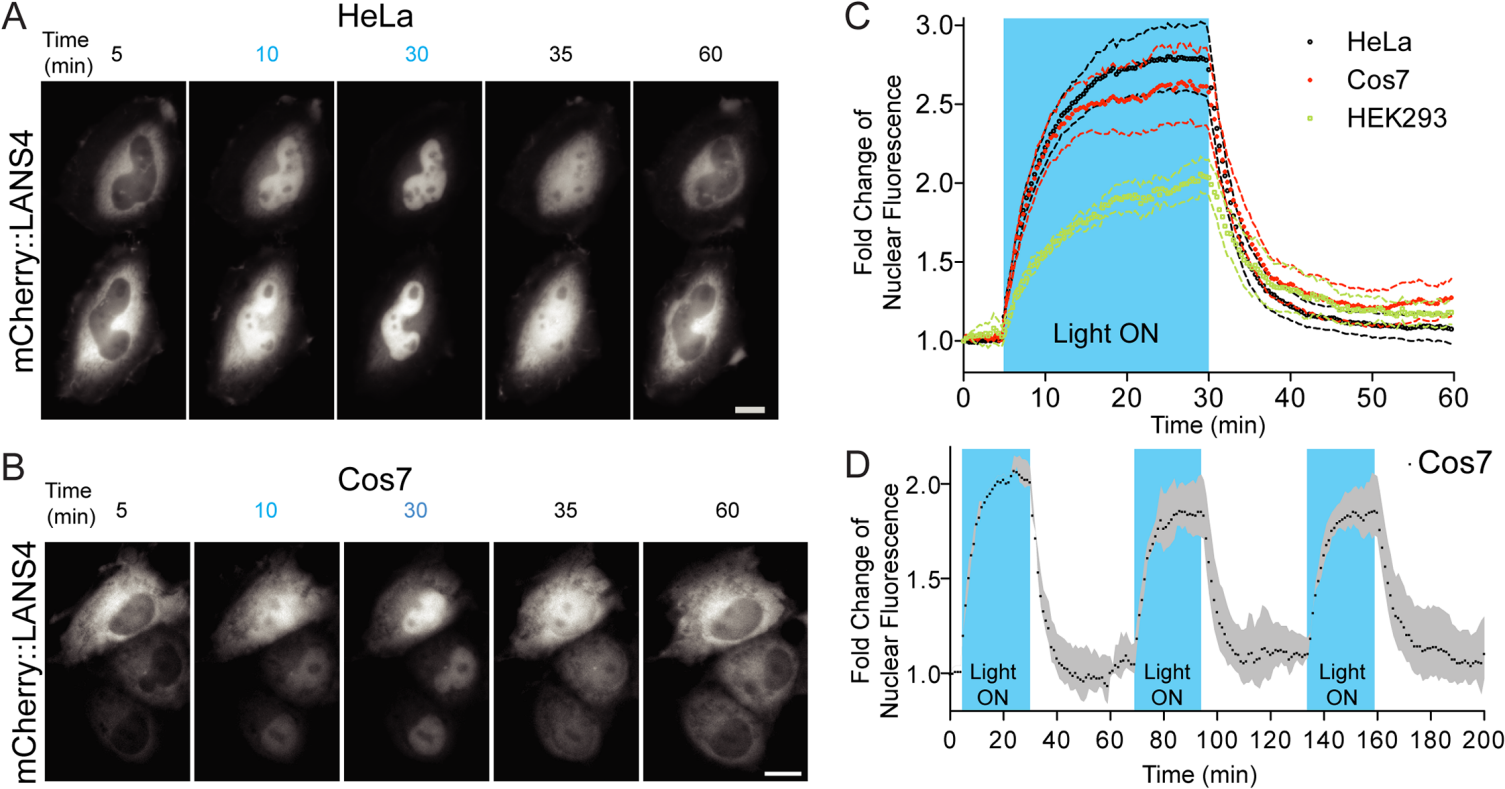
Real time light induced nuclear translocation of LANS4 in mammalian tissue culture cells. (A) Representative images for light activation and reversion in HeLa cells and Cos7 (B) (scale bar = 25 μm); (c) Plotting the fold change of nuclear accumulations in HeLa, Cos7 and HEK293 (n = 4 each, mean reported ± SEM with dashed line). See also S1, S2 and S3 Movies. The blue shaded region indicates pulsed blue light activation (see Supplemental experimental procedures). (C) Multple activation reversion cycles in Cos7 (n = 2, mean reported ± SEM with shaded grey area). The blue shaded regions indicate pulsed blue light activation.

We next sought to characterize the kinetics of nuclear import and export in response to light stimulation. We performed blue light stimulation and measured the rates of nuclear import and dark reversion for LANS4 in three types of mammalian tissue culture cells—HeLa, Cos7 and HEK293 (Fig 3A, 3B and S1–S3 Movies). Nuclear fluorescence upon activation was measured and fold changes of nuclear accumulation were fit by single exponentials with t_1/2_ = 3.3 ± 0.02 minutes for HeLa (n = 4), t_1/2_ = 2.7 ± 0.03 minutes for Cos7 (n = 3) and t_1/2_ = 5.9 ± 0.01 minutes for HEK293 (n = 5) (Fig 3C). Upon stopping the blue light stimulation, the nuclear export kinetics were similarly measured and fit, yielding t_1/2_ = 2.5 ± 0.01 minutes for HeLa, t_1/2_ = 2.8 ± 0.02 minutes for Cos7 and t_1/2_ = 3.2 ± 0.02 minutes for HEK293 (Fig 3C). The differences observed between the cell types may result from differential expression of importins and exportins [27]. Nuclear localization appeared fully reversible, with no significant loss in activation level after multiple cycles of blue light activation and reversion over the course of a few hours (Fig 3D).

### LANS enables robust transcriptional control in yeast

**Fig 4:**
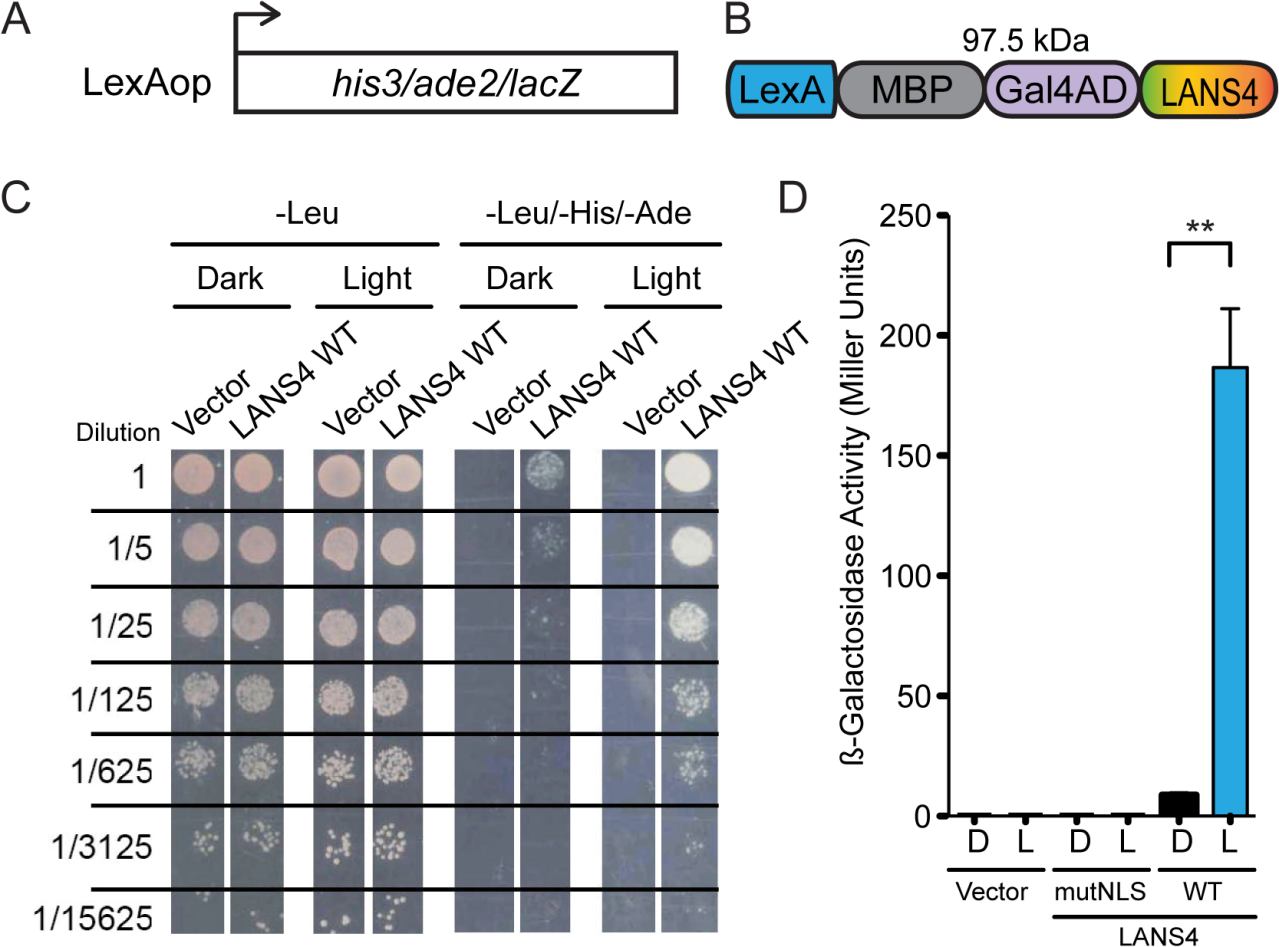
Light induced transcription via light mediated nuclear translocation in yeast. (A) NMY51 contains *his3*, *ade2* and *lacZ* genomic reporter genes under the control of LexAop. (B) Schematic of the LANS controlled artificial transcription factor in yeast (C) Growth assay of LANS controlled transcription factor in NMY51. The left panel shows growth on media lacking leucine, which confers plasmid resistance and demonstrates that the light used does not affect regular yeast growth. The right panel demonstrates light dependent growth on media lacking leucine, histidine and adenine. (D) β-galactosidase activity measurements upon blue light induced transcription activation with LANS4 n = 3 each, mean reported ± SEM and statistical significance is calculated with unpaired two-tailed t-student’s test (p = 0.0019).

To determine whether LANS could be used to control protein function in cells, we sought light-mediated control of transcription in yeast. We used the NMY51 yeast strain (Fig 4A) and an NLS reported plasmid system [28] to which we fused LANS4 to its C-terminus (Fig 4B). Colony growth assays showed light-dependent survival when grown on media lacking histidine and adenine with no background detected for the vector (Fig 4C). Normal yeast growth was not affected by blue light (Fig 4C, left panel: growth minus leucine). We then grew liquid cultures in light and dark and performed β-galactosidase assays to quantify the levels of transcriptional activation. A 21-fold change in signal was observed (8.8 ± 0.7 Miller Units (n = 3) in the dark and 187 ± 24 Miller Units (n = 3) in the light). No detectable transcription was seen for a construct with a mutated conditional nuclear localization signal where all lysines and arginines were substituted with alanines (MAAAAVALD). These data demonstrate that LANS can be used to control the activity of a transcription factor by regulating its nuclear localization.

### LANS can be used to precisely control nuclear translocation in *C*. *elegans* embryos

**Fig 5:**
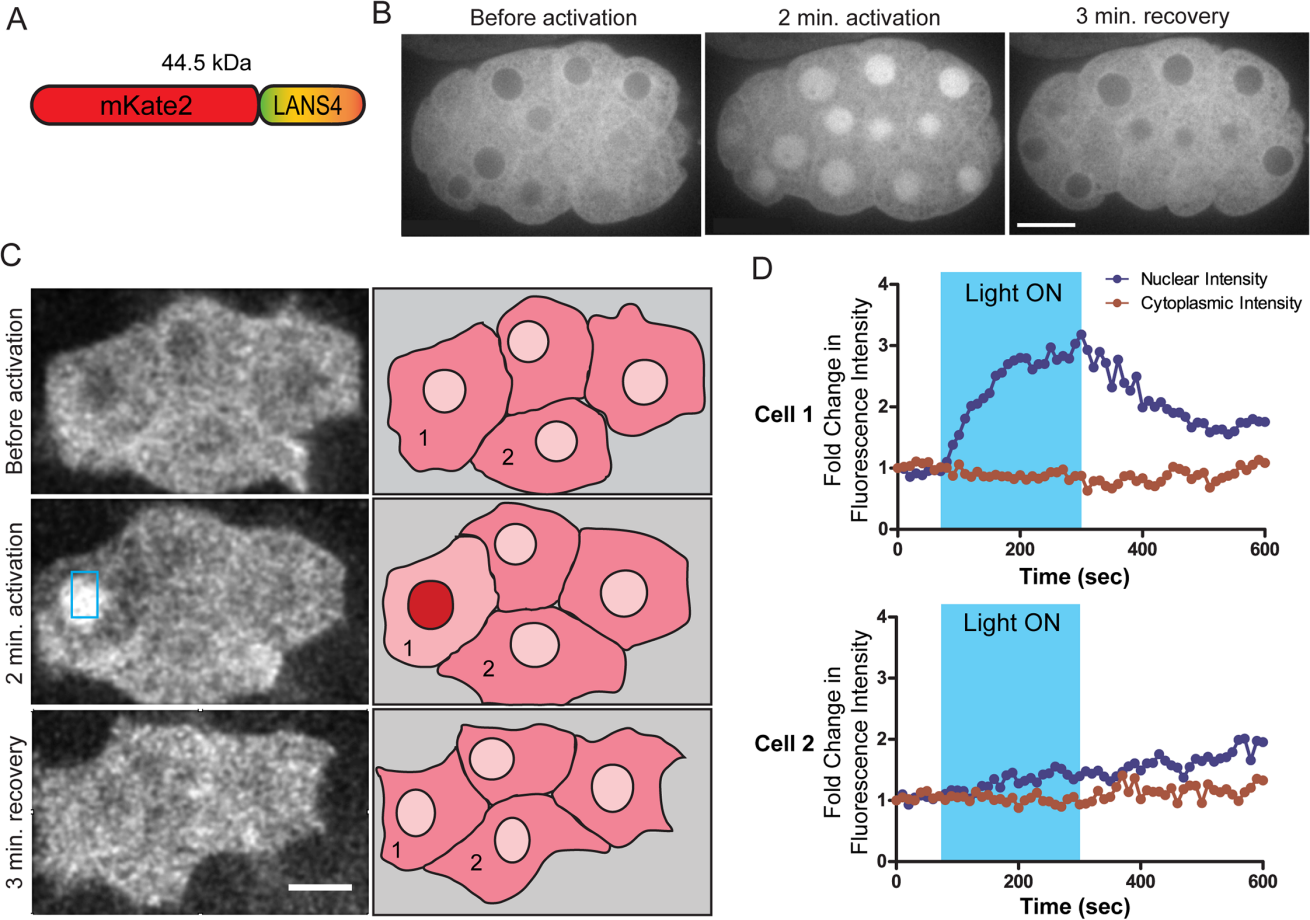
Light activated nuclear translocation in *C*. *elegans* embryo. (A) Schematic of the mKate2::LANS construct that was expressed in *C*. *elegans* embryos (B) Confocal images of an embryo expressing mKate2::LANS ubiquitously and subjected to photoactivation with blue light. Scale bars represent 10 μm. See also S4 Movie. (C) Left: Confocal images of four mKate2::LANS expressing MS lineage cells on the ventral surface of a late gastrulation-stage embryo. The blue box in the center image indicates the region that was photoactivated with blue light. Brightness and contrast were adjusted to compensate for photobleaching. Scale bar represents 5 μm. Right: Sketches summarizing the observed localization. Numbers correspond to the cell numbers in (D). See also S5 Movie. (D) Quantification of nuclear and cytoplasmic fluorescence intensities as a function of time for the two cells labelled in (C). Cell 1 was illuminated with blue light, and Cell 2 is a neighboring cell. These measurements were corrected for photobleaching (see materials and methods).

To test whether LANS can be used to regulate protein nuclear localization *in vivo*, we took advantage of the optical clarity and ease of genetic manipulation of the *C*. *elegans* embryo. We fused LANS4 to the red fluorescent protein mKate2 (Fig 5A) and expressed it in *C*. *elegans* embryos under the control of the *his-72* promoter and *tbb-2* 3’UTR. This promoter and 3’UTR support ubiquitous expression throughout development, with the strongest expression in developing embryos ([29] and D.J.D., unpublished observations). The fusion protein was cytosolic in embryos kept in the dark, but translocated rapidly (< 2 minutes) into the nucleus upon blue light activation (Fig 5B and S4 Movie). It returned fast (< 3 minutes) to the cytosol after the illumination was stopped. Expression and photoactivation of LANS did not appear to cause toxicity, since the embryos continued developing normally and hatched into viable L1 larvae after the experiment (n = 8 embryos from 2 separate experiments). We next tested whether we could achieve precise spatial control of nuclear translocation by targeting photoactivation to a single cell. For these experiments, we used embryos expressing mKate2::LANS4 in mesodermal precursors of the MS cell lineage under the control of the *ceh-51* promoter [30]. Illumination of a cell expressing mKate2::LANS4 resulted in rapid nuclear translocation, which was reversed when the illumination was stopped (Cell 1 in Fig 5C and 5D and S5 Movie). No change in mKate2::LANS4 localization was detectable in a neighbouring cell that was not illuminated (Cell 2 in Fig 5C and 5D and S5 Movie). The activation and recovery curves were well fit by single exponentials with t_1/2_ = 49 ± 9 seconds for activation and t_1/2_ = 67 ± 9 for recovery (n = 11 experiments). We conclude that LANS can be used to control nuclear localization with high temporal and spatial precision in a living *C*. *elegans*.

### LANS can be used to manipulate cell fate *in vivo*

To test whether LANS could be used to control the activity of a protein *in vivo*, we sought to manipulate the development of the *C*. *elegans* vulva, a classical model system for studying cell fate specification [31]. During the third larval stage, six vulval precursor cells with equivalent developmental potential can be induced to adopt either primary or secondary vulval fates in response to an EGF signal from the nearby anchor cell. In wild type animals, a single cell called P6.p receives the strongest EGF signal and adopts the primary vulval fate. Its neighbors, P5.p and P7.p, adopt the secondary vulval fate in response to a weaker EGF signal from the anchor cell together with a Notch signal from P6.p [31]. The remaining 3 precursor cells normally adopt non-vulval fates. Activating mutations in the EGF/Ras/Raf/MAPK signalling pathway cause ectopic induction of the primary vulval fate, resulting in a Multivulval (Muv) phenotype. Loss-of-function mutations in this pathway impair vulval induction and cause a Vulvaless (Vul) phenotype [31].

**Fig 6:**
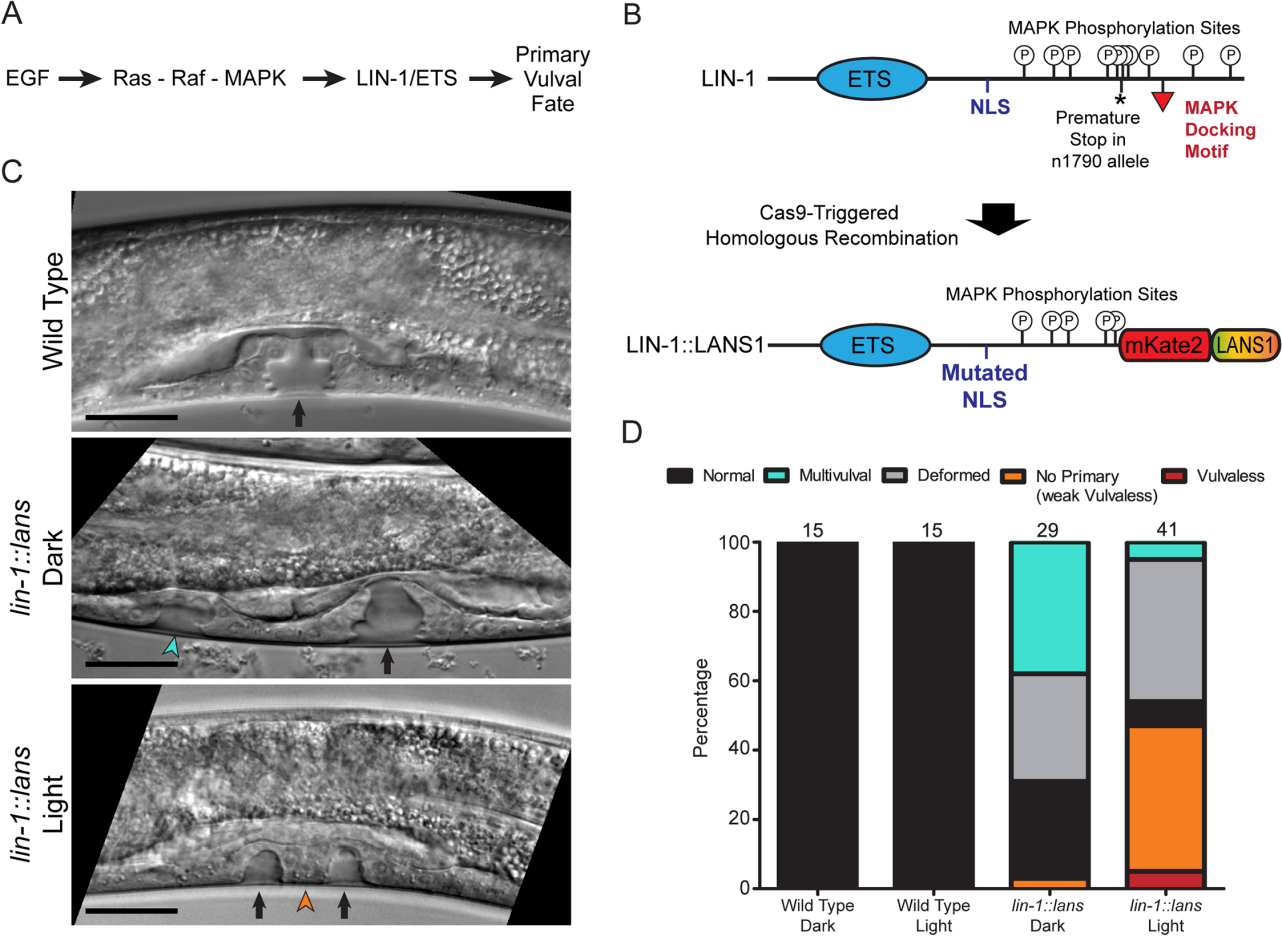
Control of vulval development via photoactivatable LIN-1. (A) Simplified schematic of the role of LIN-1 in vulval fate specification. (B) Top: Schematic of the wild type LIN-1 protein. Bottom: Schematic of the LIN-1::LANS protein produced after modification of the native *lin-1* locus using Cas9-triggered homologous recombination. See also S4 Fig. (C) DIC Images of the developing vulvae in mid-L4 larvae from the indicted strains and conditions. Top panel: Black arrow indicates the normal, symmetric vulval invagination. Middle panel: Black arrow indicates the main vulval invagination, and green arrowhead indicates an extra vulval invagination. Bottom panel: Orange arrowheads indicate small invaginations produced by the secondary vulval precursors, and black arrow indicates the plug of tissue derived from the failed primary cell. Scale bars represent 20 μm. (D) Quantification of phenotypes in the indicated strains and conditions. See Methods for detailed definitions of each phenotype. Numbers at the top of each bar indicate the total number of animals scored in this experiment. These data are from a single experiment; the experiment was repeated three times, using two independently isolated *lin-1*::*lans* alleles, with similar results.

The LIN-1/ETS transcription factor is a downstream target of the MAPK pathway and is thought to function as an inhibitor of the primary vulval fate (Fig 6A). Strong *lin-1* loss of function mutations cause all six vulval cells to adopt primary or secondary vulval fates, independent of the activity of the MAPK pathway, resulting in a strong Multivulval phenotype [32–34]. Conversely, gain of function mutations in *lin-1* result in repression of the primary vulval fate [35]. MAPK phosphorylates LIN-1 on multiple residues in its C-terminal tail (Fig 6B), which inactivates LIN-1 and allows cells to adopt the primary vulval fate [35].

To generate a light-inducible *lin-1* allele, we modified the endogenous *lin-1* gene using Cas9-triggered homologous recombination [36]. We introduced three molecular changes, with the goal of eliminating the normal regulation of LIN-1 by MAPK and replacing it with optogenetic regulation (Fig 6B and S4 Fig). First, we truncated the C-terminus, mimicking the *n1790* gain of function allele that eliminates the MAPK docking site and most of the predicted phosphorylation sites [35]. Second, we mutated a putative endogenous NLS. Third, we inserted sequence encoding mKate2::LANS1. We predicted that the resulting LIN-1::LANS1 fusion protein would be sequestered in the cytosol and inactive in the dark, but would localize to the nucleus and be constitutively active in the light.

We examined the phenotypes of *lin-1*::*lans1* animals raised in the dark or under blue light. Continuous illumination for two days had no effect on the development of wild type animals (Fig 6C and 6D and DJD, unpublished observations), indicating that the light levels used in this experiment were non-toxic. A substantial fraction (38%) of *lin-1*::*lans1* animals raised in the dark exhibited a Multivulval phenotype, consistent with our prediction that LIN-1::LANS1 should have reduced activity in the dark (Fig 6C and 6D). However, it is important to note that this phenotype is less severe and less penetrant than that of *lin-1* null alleles [32–34], suggesting that LIN-1::LANS1 retains some activity in the dark. This residual activity may be due to shuttling activity of LANS (see above), which results in the LIN-1::LANS1 transient nuclear localization even in the dark.

Illumination with blue light efficiently suppressed the Multivulval phenotype of *lin-1*::*lans1* animals (5% Muv; p = 0.001, Fisher’s exact test), indicating that LIN-1::LANS1 was activated by blue light. In addition, we observed animals that had, instead of a normal vulval invagination, a plug of tissue flanked by two smaller-than-normal invaginations (Fig 6C). We interpret this as a weak Vulvaless phenotype, in which specification of the primary cell fails, but the secondary cells still invaginate. This phenotype was observed significantly more frequently in animals raised under blue light (Fig 6D; 42% vs. 3%; p = 0.0002, Fisher’s exact test), suggesting that light-activated LIN-1::LANS1 repressed the primary vulval fate, as predicted. Consistent with our interpretation of the weak Vulvaless phenotype, a small fraction (5%) of *lin-1*::*lans1* animals raised under blue light were completely Vulvaless (Fig 6D). It is important to note that the *n1790* mutation, on which *lin-1*::*lans1* was modelled, produces a similarly mild Vulvaless phenotype, with 54% of *n1790* animals showing vulval defects [35]. The penetrance of Vulvaless / weak Vulvaless phenotypes in *lin-1*::*lans1* animals raised under blue light (54%) is similar to the penetrance of vulval defects in *n1790* animals. Taken together, these data indicate that insertion of the LANS1 coding sequence into the *lin-1* gene allowed optogenetic control of an endogenous transcription factor and manipulation of cell fate decisions in a living animal.

## Discussion

The LANS switch exhibits light dependent binding to both importin α 5 and importin α 7, indicating that multiple importin α proteins can mediate its nuclear import. Although the switch accumulates in the nucleus upon light stimulation, in the case of small proteins, passive diffusion through the nuclear pore complex results in even nuclear/cytoplasmic distribution in the dark. In order to drive the switch out of the nucleus in the dark, we coupled the switch with a constitutive nuclear export signal, which was shown to require Crm1 for its function. Modulating the strength of the nuclear export signal permits tuning the nuclear/cytoplasmic distribution of the protein of interest. The switch is further tunable using previously reported AsLOV2 dark state stabilizing mutations [37]. LANS works in a range of mammalian tissue culture cells with similar kinetics, which lead to fast nuclear import and cytoplasmic return. It remains functional after hours of cycled activation and reversion, with the protein returning to initial nuclear levels when light stimulation is stopped.

In several ways our results provide independent confirmation of the findings of Niopek et al. [13] in their use of the AsLOV2 domain to control nuclear import. Like us, they found it necessary to test different NES and NLS signals in order to maximize the dynamic range of their switch. Their best performing switches showed a ~3-fold change in cytoplasmic/nuclear protein levels, similar to what we observe. They also observed similar kinetics of nuclear import and export in mammalian cells with half times near four minutes.

We are aware of no reports of light activated transcription in *C*. *elegans* and only four studies that demonstrate light mediated transcription in multicellular organisms [38–41]. Most showed activation of specific ectopic reporter genes and required the insertion of a DNA binding region in the promoter of the gene of interest. This approach is limited to the activation of a single or a few genes at a time. Only one study demonstrates transcriptional activation of an endogenous gene in a living organism taking advantage of Cry2 and CIB1 light dimerization coupled with TALE DNA-binding domain [41]. We created a single component, simple-to-use switch, which allowed us to apply CRISPR/Cas9-triggered homologous recombination for direct, single-step control of an endogenous transcription factor and, we infer based on resulting phenotypes, its set of gene targets. Consequently, we were able to trigger a systemic cellular response and control cell fate specification in *C*. *elegans*, opening a wide range of possibilities for research.

## Materials and Methods

### DNA cloning

All cloning PCR amplifications were performed using high-fidelity Q5 polymerase and all preliminary construct screens were carried out by colony PCR using Taq polymerase. All enzymes were purchased from New England Biolabs (NEB). All plasmids were verified by DNA sequencing (GeneWiz).

### Light illumination setup

The illumination setup for the fluorescence polarization assays was as described in Lungu et al, 2012. Briefly, collimated blue LED with maximum emission at 455 nm (Thorlabs) provided 6 mW/cm^2^ illumination to a sample in a 1 cm quartz cuvette.

For the *S*. *cerevisae* and *C*. *elegans* experiments, a LED strip with maximum emission at 465 nm (Mouser Electronics, cat. #: 901-SB-0465-CT) was placed in an array 25 x 35 cm, 15 cm above the samples in an incubator set at 30°C for yeast and 20°C for *C*. *elegans*, thus obtaining even illumination of ± 500 μW/cm^2^ for yeast and ± 250 μW/cm^2^ for *C*. *elegans*.

### Protein expression

*Avena sativa* phototropin-1 gene corresponding to residues 404–546 encoding for the LOV2 domain (Uniprot: O49003) was amplified with two-step overlap extension PCR introducing the designed nuclear localization signal and clone in pQE-80L using BamHI and HindIII restriction digest. The resulting plasmid was transformed in BL21 (DE3) pLysS and bacteria grown in 1.5 L Luria Bertani media till reaching OD_600_ ~ 0.6 upon which protein production was induced with 500 mM IPTG overnight (18-20h) at 25°C. Cell pellets were resuspended in 50 mM Tris pH 7.5, 1 M NaCl, 10 mM Imidazole, 10 mM β-ME and 1 mM PMSF and sonicated for 15 mins (5 sec. ON, 5 sec. OFF) on ice at 4°C. Lysed cells were then centrifuged for 30 min at 18564 RCF, then the supernatant filtered through 5 μM filter and loaded on 5 mL HisTrap IMAC columns (GE Healthcare). The columns were washed with 30 CV of 50 mM Tris pH 7.5, 1 M NaCl, 10 mM Imidazole, 10 mM β-ME and eluted on BioLogic LP (BioRad) using a gradient against 50 mM Tris pH 7.5, 100 mM NaCl, 500 mM Imidazole, 10 mM β-ME. The eluted protein was concentrated with Amicon Ultra-15 (Millipore) to 2 mL and subjected to size exclusion chromatography in 50 mM Tris pH 7.5, 100 mM NaCl and 1 mM DTT with HiLoad 16/600 Superdex 75 (GE Healthcare) run on Akta FPLC (Amersham). Importins were expressed and purified in the same way as described above. After IMAC the proteins were concentrated to 5 mL, dialyzed against 5 L of 50 mM Tris pH 7.5 overnight at 4°C and purified over HiTrap Q HP column (GE Healthcare) eluted over a gradient against 50 mM Tris pH 7.5 and 1 M NaCl. Then, the protein was were concentrated to 5 mL and subjected to size exclusion chromatography in 50 mM Tris pH 7.5, 100 mM NaCl and 1 mM DTT with HiLoad 16/600 Superdex 200 (GE Healtchare). All protein concentrations were determined using the BCA assay following manufacture protocol (Thermo Scientific Pierce).

### Fluorescence polarization

Peptide of the following sequence GDMAAKRVKLD was synthesized at UNC- Chapel Hill and amine labeled using 5- and 6-Carboxytetramethylrhodamine (TAMRA) dye. Peptide concentration was determined by measuring absorbance of the TAMRA dye at 555 nm using 65,000 M^-1^ cm^-1^ as extinction coefficient. All fluorescence polarization measurements were performed on FluoroMax3 (Jobin Ybon Horiba) fluorescence spectrometer using 25 nM of the TAMRA labelled peptide in 3 mL of 50 mM Tris pH 7.5, 100 mM NaCl and 1 mM DTT. The dye was excited with polarized light at 555 nm and polarization of emitted light measured at 584 nm. The sample was held in a 1 cm quartz cuvette at 25°C, increasing concentrations of importin α were titrated and two polarization values for dark and lit state binding were recorded for each titration point. The polarization value for the lit state binding was collected after 2 minutes of blue light illumination and for the dark state by another measurement taken after 5 minutes in absence of light for the same titration point. Finally, the competition was fit for one-site total binding with Prism 5 (GraphPad) and IC50 values were used to determine the Kd with the online calculator [42].

### Mammalian cell culture

HeLa, Cos7 HEK293T (ATCC) tissue cultures were grown at 37°C, 10% CO_2_ in DMEM supplemented with 10% (v/v) HyClone Standard Fetal Bovine Serum (Thermo Scientific) and 1% (v/v) GlutaMAX (GIBCO). Cells were passaged every 2–3 days usually in Nunc T-25 or T-75 culture flasks (Thermo Scientific).

### Mammalian cell imaging

Constructs consisting of mCherry and AsLOV2cNLS with or without NES signals located between mCherry and AsLOV2cNLS were cloned in pTriEx with restriction digest cloning using NcoI and HindIII restriction sites. All constructs were sequence verified.

Coverslips were washed with PBS (GIBCO) and coated with 10 μg/ml fibronectin at room temperature for a minimum of 1.5 hours. Cells were seeded for a minimum of 3 hours to overnight, then transfected using FuGENE 6 (Promega) and imaged after about 18 hours post-transfection in Ham’s F-12K medium free of Phenol red (Caisson) and containing 10% FBS buffered with 10 mM HEPES pH 8. Coverslips were mounted in an Attofluor live cell chamber (Invitrogen) placed in a microscope stage with a heated stage adaptor (Warner) and an objective temperature controller (Bioptechs).

An Olympus DSU-IX81 Spinning Disk Confocal coupled with Andor solid-state lasers (Andor) was used to perform the nuclear export signal screen. Z-stacks of 12 μm at 0.5 μm steps were acquired with a PlanApo 60× objective (Oil, N.A. 1.42) using a 561 nm laser set at 20% intensity (150 EM gain and 300 ms exposure).

Live cell timelapse series were collected with an Olympus IX81 epifluorescence microscope equipped with a ZDC focus drift compensator and a Photometrics CoolSnap ES2 CCD camera (Roper Photometrics). A UPlanFLN 40× objective (Oil, N.A. 1.30) was used with an ET572/35x filter for mCherry detection and 1% (UVND 2.0, ET430/24x) for blue light activation of LANS.

### Yeast transcription

LANS4 was amplified and cloned into pNIA-CEN-MBP plasmid [28] using EcoRI and BamHI restriction sites. The resultant plasmid was transformed via high efficiency lithium acetate transformation [43] and plated on SC-Leucine droupout agar plates.

Survival assays were performed as follows: Fresh colonies were grown overnight at 30°C in 5 ml SC-Leucine. On the next day, the cell density was measured at OD_600_ and cultures diluted in 200 μl of OD_600_ = 1, followed by 8 5-fold serial dilutions. Then, 5 μl of each of the dilutions were pipetted and spotted using a multichannel pipette (Gilson) onto respective dropout plates. The dark condition plates were wrapped in aluminium foil and placed in the same incubator as the lit condition at 30°C. Blue light (465 nm) at 500 μW/cm^2^ was provided with LED strip lights attached at the incubator (look at illumination settings). Yeast plates were scanned after 72h incubation using an HP ScanJet 4850 scanner and resulting images were cropped and arranged using Adobe Photoshop.

β-Galactose assay were performed as follows: Fresh colonies were grown overnight at 30°C in 5 ml SC-Leucine. On the next day, the cell density was measured at OD_600_ and 2 mL cultures were diluted to OD_600_ = 0.2 in duplicates—one for a light and another for a dark condition (falcon tubes were wrapped in aluminium foil). Cultures were grown at 30°C in a shaking incubator (250rpm) till reaching OD_600_ ± 0.8 in presence or absence of blue light (465 nm) at 500 μW/cm^2^ via LED strip light wrapped around the tube rack. The resulting cultures were pelleted in triplicates and β-Galactose assay using CPRG for a substrate was performed according to the manufacturer (Clontech).

### *C*. *elegans* culture and strain construction

S1 Table lists the *C*. *elegans* strains used in this study including the native N2 [44]. All strains were maintained at 20°C or 25°C on NGM medium, using *E*. *coli* strain OP50 as a food source. To express mKate2::LANS4 ubiquitously in early embryos, an mKate2::LANS4 coding sequence was designed using optimal *C*. *elegans* codons and synthetic introns [45]. This sequence was assembled from gBlocks gene fragments (Integrated DNA Technologies) using Gibson assembly (New England BioLabs) and cloned into a modified pCFJ352 vector [46] containing the *his-72* promoter and *tbb-2* 3’UTR. This construct was inserted as a single-copy transgene into the *ttTi4348* locus on LG I using MosSCI [46]. To express mKate2::LANS4 in MS cells, a construct carrying the mKate2::LANS coding sequence was cloned into a modified pCFJ151 vector [46] containing the *ceh-51* promoter and *tbb-2* 3’UTR. This construct was injected into wild type worms (strain N2) to generate extrachromosomal arrays [47].

To insert LANS1 into the *lin-1* locus, we used Cas9-triggered homologous recombination [36]. S4 Fig shows a schematic of our gene targeting strategy. We modified the Cas9–sgRNA expression plasmid pDD162 by inserting the guide sequence ATGACGTCGTGGAGGGCGATA. Next, we constructed a homologous repair template by first cloning a 3.9 kb genomic fragment, comprising the last 3.3 kb of the *lin-1* gene followed by a 0.2 kb intergenic region and the first 0.5 kb of the downstream *M70*.*5* gene, into the pCR-Blunt TOPO vector. We made 4 modifications to this construct: 1) We deleted a 1 kb fragment comprising the last 270 bp of *lin-1* coding sequence and an intervening intron, and replaced this fragment with the mKate2::LANS1 coding sequence. 2) We mutated the putative NLS sequence RQCRKRSL to AQCAAASL. 3) We inserted a selection cassette, comprising a hygromycin resistance gene [48] followed by *unc-58(n495)*, into the intergenic region downstream of the *lin-1* 3’UTR. We intended to use the dominant Unc-58 phenotype as a visible marker for the *lin-1* allele, but animals generated using this construct do not have an Unc-58 phenotype, indicating that, although *unc-58(n495)* is a dominant mutation, insertion of a single extra copy of *unc-58(n495)* into an *unc-58(+)* background is not sufficient to cause the Unc-58 phenotype. 4) Finally, because *lin-1* and *M70*.*5* are close together and located on the same strand, it is possible that the *lin-1* 3’UTR contains sequences that function in the promoter of *M70*.*5*, and that insertion of the selectable marker in the intergenic region between *lin-1* and *M70*.*5* could disrupt *M70*.*5* expression. To avoid this, we inserted a second copy of the *lin-1* 3’UTR downstream of the selectable marker. The sequence of the final homologous repair template is available upon request.

The *elk-2* gene of *C*. *elegans* has >90% nucleotide sequence identity to the region of *lin-1* that we wished to modify, which made it impossible to identify a Cas9 target site that was unique to *lin-1*. Therefore, to avoid inadvertently modifying *elk-2* instead of *lin-1*, we performed Cas9-triggered homologous recombination in the parent strain VC2110, which carries a deletion in *elk-2* that removes part of the region homologous to *lin-1*, including the Cas9 target site that we selected. An injection mix containing 50 ng/μL Cas9–sgRNA plasmid, 10 ng/μL homologous repair template, and co-injection markers [36] was injected into young VC2110 adults. The injected animals were allowed to lay eggs for 3 days at 25°C, and then hygromycin was added to the plates to a final concentration of 0.5 mg/mL to kill non-transformed animals. After 7 days of selection, the plates were heat shocked for 4h at 34°C to kill animals carrying extrachromosomal arrays (via PEEL-1 negative selection [46]). Candidate *lin-1*::*lans* animals that survived both rounds of selection were singled to establish lines, and correct insertion of *lans* was confirmed by PCR. We then outcrossed to remove the unlinked *elk-2* mutation that facilitated strain construction.

### Photoactivation experiments in *C*. *elegans* embryos

Embryos expressing mKate2::LANS4 were mounted on polylysine-coated coverslips and gently flattened using 2.5% agar pads. For whole-embryo photoactivation experiments (Fig 5B), we used a Nikon Eclipse Ti microscope equipped with a 100X, 1.49 NA objective and a Yokagawa CSU-X1 spinning disk head and controlled using Metamorph (Molecular Devices). Confocal images of mKate2 fluorescense were captured every 10s, and the embryos were illuminated with ~180 μW/cm^2^ of blue light from a 488 nm laser for 5s between each pair of image acquisitions (i.e., 50% photoactivation duty ratio). For single-cell photoactivation experiments (Fig 5C), we used a Zeiss LSM710 laser scanning confocal microscope equipped with a 100X, 1.3 NA objective and controlled by Zeiss Zen software. Images were acquired at 10s intervals, and photoactivation was done between each pair of acquisitions by scanning a 458 nm laser at ~170 W/cm^2^ over a region of interest defined using the FRAP/photoactivation function of the Zen software. Each pixel was illuminated for a total of 63 μs during photoactivation.

To measure mKate2::LANS4 nuclear localization, we made line scans across a region of the image that encompassed the nucleus and cytoplasm of each cell of interest. Pixel intensities were measured from kymographs and converted to fluorescence intensity by subtracting off-embryo background. To correct for photobleaching, we normalized each measurement to the total integrated fluorescence intensity of the entire image.

### Scoring of vulval phenotypes

Animals were synchronized by allowing embryos to hatch in the absence of food and arrest as L1 larvae. Synchronized larvae were plated on NGM plates seeded with *E*. *coli* OP50, and the plates were placed under blue LED illumination (see above). Dark controls were placed in the same incubator and wrapped in aluminium foil to prevent light exposure. When the animals reached mid-L4 stage (approximately 48 hours), they were mounted on 2.5% agar pads containing 10 mM sodium azide as a paralytic. DIC images of the developing vulva were acquired using a Nikon Eclipse E800 microscope equipped with Nomarski optics and a 100X, 1.4 NA objective. Animals were scored as Normal if there was a single, symmetric vulval invagination; as Multivulval if they had at least one secondary invagination in addition to the main vulva; as “weak Vulvaless” if, instead of a fully formed vulval invagination, there were two small, equally-sized invaginations separated by apparently undifferentiated tissue; as Vulvaless if there was no vulval invagination; and as Deformed if there was a single vulval invagination that was misshapen or asymmetric, or if the morphology was too abnormal to be confidently assigned to one of the other categories. Data were plotted and p values were calculated using Graphpad Prism software.

## Accession codes

Addgene: pTriEx-mCherry::LANS1, 60784; pTriEx-mCherry::LANS4, 60785.

## Supporting Information

S1 Fig LANS computational design. (A) Rosetta model of the designed NLS on yeast karyopherin (B) Rosetta model of the chimeric AsLOV2cNLS.
(TIF)

S2 Fig Biophysical characterization of LANS. (A) Peptide binding to importin α 5 and (B) importin α 7 (C) AsLOV2 native sequence does not compete with an NLS for binding to α 5 and α 7 in neither dark nor light conditions (D) Fusing the Myc NLS directly at the C-terminus of AsLOV2 (PDB: 2v0u) residue 546 leads to tight but light independent binding to importin α 5 with affinities measured at about 1 nM. (E) AsLOV2cNLS chimera preserves wild type AsLOV2 reversion kinetics.
(TIF)

S3 Fig LANS is actively exported and tolerates small and large C-terminal fusions. (A) Treatment with 200 nM of Leptomycin B for 10 minutes reverts the nuclear/cytoplasmic distribution of LANS with NES signals to one without a nuclear export signal. Confocal microscopy with a single nuclear optical slice (scale bar 15 μm) (B) C-terminal fusion of a short peptide (flag tag) and large globular protein (MBP) constructs. (C) Epifluorescent microscopy for flag tag and MBP in the dark and after 10 minutes of blue light activation (scale bar 25 μm).
(TIF)

S4 Fig Cas9-triggered homologous recombination schematic. Schematic of our strategy for modifying the *lin-1* locus using Cas9-triggered homologous recombination. See Experimental procedures for details.
(TIF)

S1 Movie mCherry::LANS4 blue light activation and reversion in HeLa. Still images from this movie are shown in Fig 4A. The scale is provided with a scale bar = 25 μm, the time is shown as min:sec and “ON” indicates intermittent blue light activation (see Supplemental experimental procedures).
(MOV)

S2 Movie mCherry::LANS4 blue light activation and reversion in Cos7. Still images from this movie are shown in Fig 4B. The scale is provided with a scale bar = 25 μm, the time is shown as min:sec and “ON” indicates intermittent blue light activation (see Supplemental experimental procedures).
(MOV)

S3 Movie mCherry::LANS4 blue light activation and reversion in HEK293. The scale is provided with a scale bar = 25 μm, the time is shown as min:sec and “ON” indicates intermittent blue light activation (see S1 Text).
(MOV)

S4 Movie Whole-embryo photoactivation of ubiquitously expressed mKate2::LANS4. Still images from this movie are shown in Fig 5B.
(MOV)

S5 Movie Photoactivation of mKate2::LANS4 in a single MS cell. Still images from this movie are shown in Fig 5C.
(MOV)

S1 Table *C. elegans* strains used in this study. (XLSX)

S1 Text Supporting Materials and Methods. (PDF)
