# Supplementary figures and images for: Control of Protein Activity and Cell Fate Specification via Light-Mediated Nuclear Translocation
Source: PLoS One. 2015 Jun 17;10(6):e0128443. doi: 10.1371/journal.pone.0128443 (PMC4471001; doi:10.1371/journal.pone.0128443)

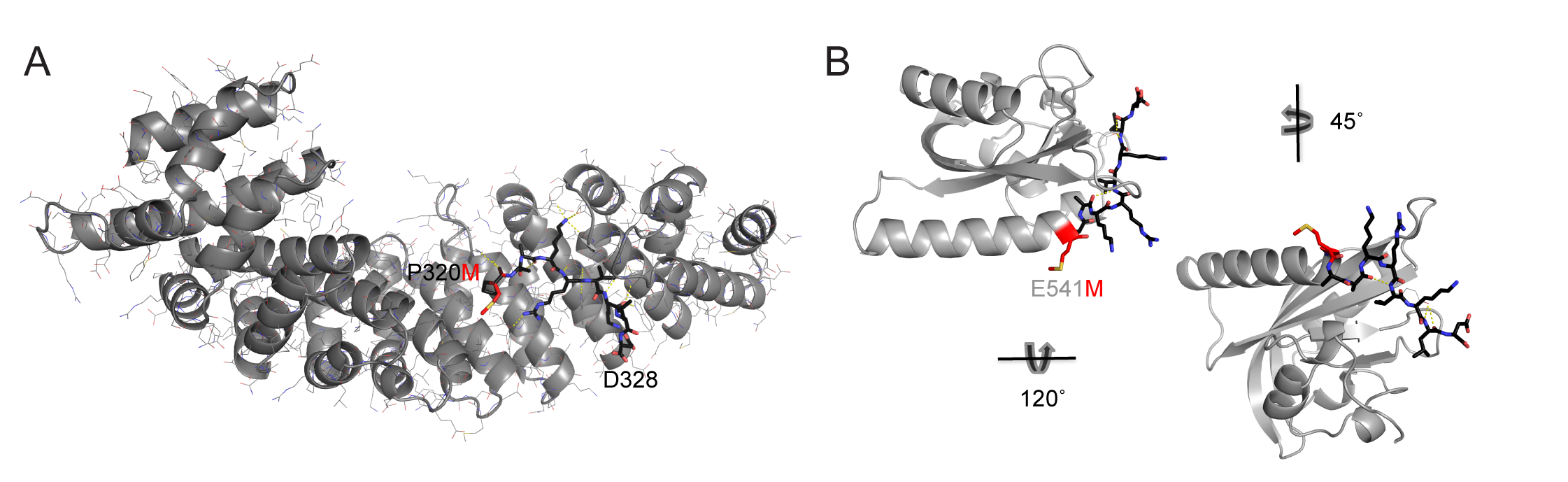

Supplement: S1 Fig — (A) Rosetta model of the designed NLS on yeast karyopherin (B) Rosetta model of the chimeric AsLOV2cNLS. (TIF) [file pone.0128443.s001.tif]

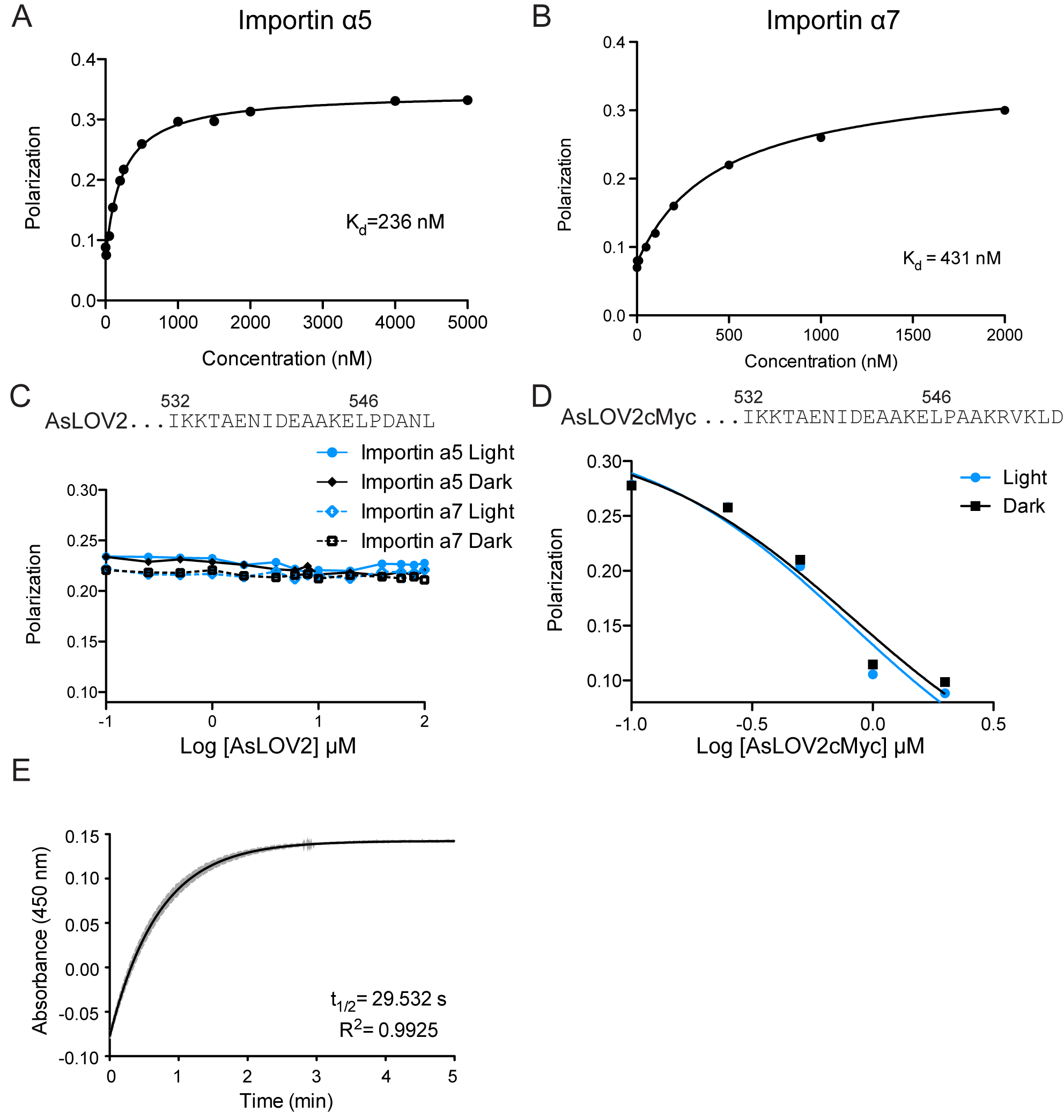

Supplement: S2 Fig — (A) Peptide binding to importin α 5 and (B) importin α 7 (C) AsLOV2 native sequence does not compete with an NLS for binding to α 5 and α 7 in neither dark nor light conditions (D) Fusing the Myc NLS directly at the C-terminus of AsLOV2 (PDB: 2v0u) residue 546 leads to tight but light independent binding to importin α 5 with affinities measured at about 1 nM. (E) AsLOV2cNLS chimera preserves wild type AsLOV2 reversion kinetics. (TIF) [file pone.0128443.s002.tif]

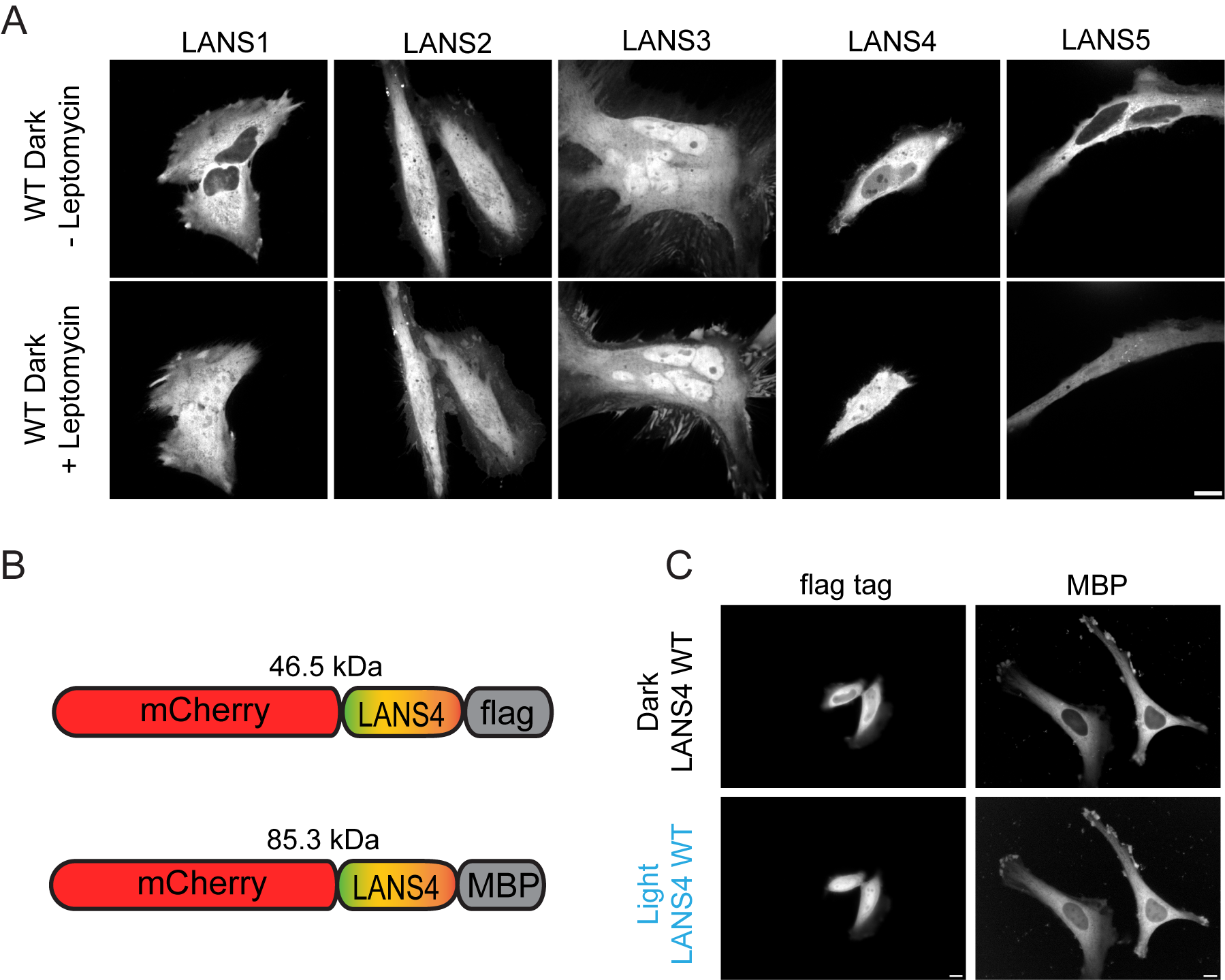

Supplement: S3 Fig — (A) Treatment with 200 nM of Leptomycin B for 10 minutes reverts the nuclear/cytoplasmic distribution of LANS with NES signals to one without a nuclear export signal. Confocal microscopy with a single nuclear optical slice (scale bar 15 μm) (B) C-terminal fusion of a short peptide (flag tag) and large globular protein (MBP) constructs. (C) Epifluorescent microscopy for flag tag and MBP in the dark and after 10 minutes of blue light activation (scale bar 25 μm). (TIF) [file pone.0128443.s003.tif]

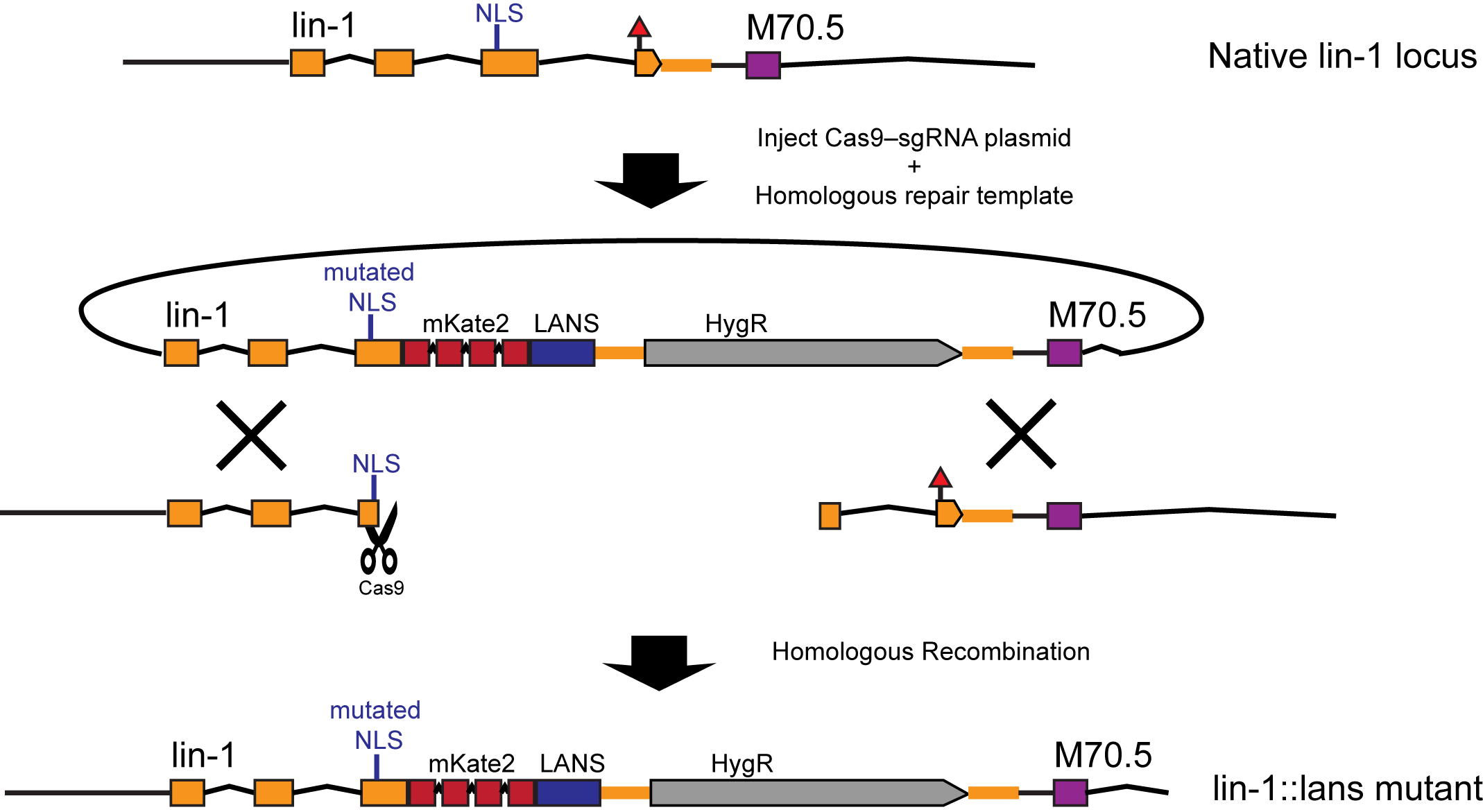

Supplement: S4 Fig — Schematic of our strategy for modifying the lin-1 locus using Cas9-triggered homologous recombination. See Experimental procedures for details. (TIF) [file pone.0128443.s004.tif]
