# Supplementary material for: Control of Protein Activity and Cell Fate Specification via Light-Mediated Nuclear Translocation
Source: PLoS One. 2015 Jun 17;10(6):e0128443. doi: 10.1371/journal.pone.0128443 (PMC4471001; doi:10.1371/journal.pone.0128443)
Supplement: S1 Text — (PDF) [file pone.0128443.s011.pdf]

Supporting materials and methods

Protein design

Chain A and C from the yeast karyopherin α structure in complex with Myc NLS (PDB: 1ee4) were extracted and used as an input to fixed backbone design with the Rosetta modelling suite. Two separate experiments (NATAA and NATRO) were carried out with 100 trajectories each restricting only the variation of amino acid at position 320 (Proline) in chain C, to any amino acid (ALLAwc). All the design trajectories converged to Methionine. The designed Myc NLS was aligned at the C-terminus of Jα in the LOV2 domain from *Avena sativa* (PDB: 2v0u). Fixed backbone design was used to mutate position 541, 545 and 546 to M, R and V correspondingly, restricting the remaining amino acid to native rotamers with NATRO. Next, the domain assembly protocol was used to fuse the residues KLD to the C-terminus of the model obtained from the previous step. Finally, the resulting model was relaxed with the Rosetta fast relax protocol and evaluated using PyMOL (Schrödinger).

Thermal reversion

Thermal reversion kinetics was determined on 20 µM of purified AsLOV2cNLS in 1100 µL of 50 mM Tris pH7.5, 100 mM NaCl and 1mM DTT after illumination for 2 min with blue light (455nm, 6.0 mW/cm^2) at room temperature. A spectrum reading was taken immediately after the illumination for every 5 nm between 500-400 nm (speed 4800 nm/min) using Cary 50 UV-Vis spectrometer (Varion). The absorbance was collected continuously for 5 minutes. The procedure was repeated 5 times for obtaining standard deviation.

Mammalian cells image acquisition and processing

Nuclear/cytoplasmic fluorescence ratios were acquired by selecting two 0.5 µm nuclear optical slices, substracting background levels using statistical correction with MetaMorph (Molecular

Devices) software. Nuclear and cytoplasmic regions were then manually selected on the average intensity projection for each image. Average intensity fluorescence normalized for the selected area was used for calculating nuclear/cytoplasmic ratios.

Single activation and reversion timelapse series were attained using a custom journal, acquiring a frame every 20 seconds, where 1-15 frames were obtained in the absence of blue light, frames 16-90 were collected with 5 sec blue light illumination, and 91-181 were collected in the absence of blue light activation. Multiple cycle of activation were performed similarly, except that a frame was collected once every 60 seconds. An image for shading correction was collected for both DIC and mCherry as the average of 10 frames. MetaMorph (Molecular Devices), was then used to perform shading correction and subtract background. Finally, the images were thresholded and corrected for bleaching using a custom MatLab script. A nuclear region of interest was then used for plotting nuclear fluorescence fold change over time.
